# A Comparative Study Between Intranasal and Intravenous Dexmedetomidine and Hemodynamic Responses During Endotracheal Intubation

**DOI:** 10.7759/cureus.35196

**Published:** 2023-02-19

**Authors:** Padmasree M.K, Kiran Nelamangala

**Affiliations:** 1 Anaesthesiology, Sri Devaraj Urs Medical College, Kolar, IND

**Keywords:** dexmedetomidine, intravenous, intranasal, premedication, hemodynamic responses, intubation

## Abstract

Introduction: Tracheal intubation and laryngoscopy may cause sympathetic stimulation, which can cause tachycardia and hypertension. To abolish the pressor response to laryngoscopy and endotracheal intubation, many medication combinations have been tried with varying degrees of efficacy.

Materials and methods: This randomized comparative study was double-blinded and included 106 subjects. Patients including those aged 18-60 belong to the American Society of Anesthesiologists (ASA) 1 and 2. These subjects were divided into two study groups. Group A received dexmedetomidine 0.5mcg/kg (200mcg diluted in 50ml syringe with normal saline (NS) up to 50cc 4mcg/ml) through an infusion pump over 40min before induction. Group B received dexmedetomidine intranasally (1mcg/kg) in undiluted which is prepared from parental preparation (100mcg/ml) and an equivalent dose of NS to the other group. The intranasal drug was dripped into both nostrils in equal volume using a 1ml syringe in a supine head-down position about 40min before induction. Both groups received an intravenous placebo and an intranasal placebo with normal saline.

Results: In our study, intranasal and intravenous groups were compared. There was no statistically significant difference in hemodynamic variables like heart rate (HR), systolic blood pressure (SBP), diastolic blood pressure (DBP), and mean arterial pressure (MAP) between the two groups (majority p value >0.05). Hence both routes can be preferred for attenuation of pressor responses.

Conclusion: Study findings demonstrate dexmedetomidine can be utilized as a premedication to lessen hemodynamic surges during endotracheal intubation with more or less the same efficacy via intranasal and intravenous routes. This result could be attributable to the fact that both intravenous and intranasal dexmedetomidine stop central catecholamine levels from rising.

## Introduction

Various hemodynamic alterations are related to the induction of General Anesthesia (GA), laryngoscopy, tracheal intubation, and extubation. Tracheal intubation and laryngoscopy may cause sympathetic activation, which can cause tachycardia and hypertension. Therefore, it's important to adequately optimize the hemodynamic responses. To lessen these sympathetic responses many medication combinations have been tried with varying degrees of efficacy [1].

Premedication is typically used to relieve anxiety, facilitate easier parental separation in children, and alleviate the need for anesthesia. Sedative, analgesic, antisialagogue, and anxiolytic qualities are desired in premedication. It should ideally have a short half-life, a quick onset, be non-parentally administered, and have no negative effects on hemodynamics [2]. Dexmedetomidine doesn’t have properties like respiratory depression because it is short-acting alpha 2 agonist and is highly selective, it got effects like analgesic, sedative effect, and anxiolytic effect. Prior to receiving anesthesia, it is the ideal medication for reducing anxiety or trepidation. It is acknowledged that dexmedetomidine was given intravenously (IV) before surgery can effectively lower the laryngoscopic stress response [3]. It's possible that dangerous hemodynamic aftereffects like reduced heart rate (HR), lowered blood pressure values, and even cardiac arrest have been recorded. Due to its sedative effect, IV dexmedetomidine has delayed recovery [4]. Alternative delivery methods, rather than rapid IV delivery, have been proposed as a way to lessen the side effects of dexmedetomidine.

Dexmedetomidine is also efficacious when administered orally, intramuscularly, and intranasal (IN). Compared to other methods, IN delivery is more practical and efficient [5]. Dexmedetomidine administered intranasally has been found to be well-tolerated by patients. IN dexmedetomidine premedication as an alternative to conventional premedication has recently been shown to have positive perioperative outcomes in multiple studies in the pediatric age group [5,6]. As far as we are aware, there has only been a small amount of research that has evaluated the effectiveness of preoperative IV dexmedetomidine with IN dexmedetomidine for attenuating hemodynamic responses during laryngoscopic intubation. Hence, to evaluate the effect of dexmedetomidine in both IN and IV routes this study has been conducted. The objectives of this study were to compare hemodynamic variables like mean arterial pressure (MAP), HR, and systolic and diastolic blood pressure in IN and IV routes of dexmedetomidine.

This article was previously presented as a paper at the ISACON 2022, Shillong on November 27, 2022.

## Materials and methods

After receiving the approval of the Institutional Ethics Committee NO.DMC/KLR/IEC/512/2022-23, 106 patients were included in a study using a randomized comparative study with double blinding. American Society of Anesthesiologists'-physical status I and II patients and age groups between 18 and 60 years were the inclusion criteria in this study after obtaining written informed consent from patients posted for elective surgeries under GA. Exclusion criteria for this trial included those with a known allergy or hypersensitivity to dexmedetomidine, known cardiac and respiratory disorders, a predicted difficult airway, nasal polyps, ulcers, and nasal septum deviation.

Prior to surgery, all patients were evaluated, investigations were reviewed, the anesthetic technique was explained, and informed consent was obtained. Patients were premedicated with Tab. Alprazolam 0.5mg and Tab. Ranitidine 150mg and fasting were strictly enforced. Patients were assigned into groups at random using computer-generated randomization after baseline measurements of their HR, non-invasive blood pressure (NIBP), and peripheral capillary oxygen saturation were taken. Forty minutes prior to induction, Group A: IV group received an IV dose of dexmedetomidine 0.5mcg/kg (200mcg diluted in a 50ml syringe with normal saline (NS) 4mcg/ml). While Group B: IN group received dexmedetomidine intranasally (1mcg/kg) undiluted from the parental solution (100mics/ml). IN drug was dripped into both nostrils with the same volume using a 1ml syringe about 40 minutes before induction in a supine head-down position. In order to maintain blinding, both groups received placebo NS of equivalent groups via IV and IN routes. The patient was taken to the operation theatre (OT) where their baseline HR, NIBP, and SPO_2_ monitoring were continued after the study drug had been administered for 40 minutes. Patients were premedicated with Inj. Glycopyrrolate 0.005mg/kg and Inj. Fentanyl 2mcg/kg before the induction and preoxygenated for 3 minutes with 100% oxygen. Inj. propofol (2mg/kg) was used to induce anesthesia, Inj. succinylcholine (2mg/kg) was used as a muscle relaxant and tracheal intubation was done using oral endotracheal tubes. Maintenance of anesthesia was done by 60% nitrous oxide in oxygen, isoflurane, and Inj. Vecuronium 0.1mg/kg as muscle relaxant. Isoflurane concentration was titrated based on the requirement. The patient was mechanically ventilated to maintain ETCO_2_ (end-tidal carbon-di-oxide) between 30 and 35mm of Hg. HR, systolic blood pressure (SBP), diastolic blood pressure (DBP), and MAP were recorded immediately and 1 min after intubation and then at 3 min, 5 min followed by at every 10 min intervals till 40 minutes post intubation. Bradycardia was treated by Inj. Atropine at 0.02mg/kg and hypotension was treated by titrating isoflurane concentration or by the rate of infusion of IV fluids. Infusion of the study drug was stopped and isoflurane was discontinued 10 mins prior to reversal. The residual neuromuscular blockade was reversed with Inj. Neostigmine 0.05mg/kg and Inj. Glycopyrrolate 0.01mg/kg. After observing the motor recovery and spontaneous breathing efforts, the patient was extubated after thorough oral suctioning.

Statistical analysis

The sample size was calculated based on a mean difference in MAP of 6% with a 95% confidence interval, an alpha error of 5%, and a 10% dropout as reported in a study by Niyogi et al., and the sample size is estimated as 53 in each group [7].

With the help of an independent t-test and chi-square test, repeated analysis of variance (ANOVA) measures the numerical variables between the two groups were compared.

## Results

In Table 1, the mean age of the IV group was 38.59 ± 11.61 years, and IN group was 40.95 ± 12.89 years. The mean age difference was statistically non-significant.

**Table 1:** Mean Age Difference between Intravenous and Intranasal

Group	Mean age	Standard deviation	P value
Intravenous	38.59	11.61	0.326 (Non-significant)
Intranasal	40.95	12.89	

In Table 2, the mean HR at baseline in the IV group was 76.41 ± 9.91 bpm, in IN group it was 71.65 ± 7.32 bpm. After post-intubation, the mean HR in the IV group was 70.1 ± 8.77 bpm, and in IN group it was 69.38 ± 7.49 bpm. The mean HR difference at baseline between the two groups was statistically significant.

**Table 2:** Difference in Heartrate between Intranasal and Intravenous S.D - standard deviation

Heart rate	Intravenous		Intranasal		P value
	Mean	S.D	Mean	S.D	
At baseline	76.41	9.91	71.65	7.32	0.006*
After post-induction	70.10	8.77	69.38	7.49	0.651

In Table 3, the difference in mean SBP between IV and IN groups at different time intervals was shown. Of them, only at 40 minutes, at intubation, and at 10 minutes post-intubation mean SBP difference between the groups was found to be significant.

**Table 3:** Difference in SBP between Intranasal and Intravenous SBP - systolic blood pressure S.D - standard deviation

SBP	Intravenous		Intranasal		P value
	Mean	S.D	Mean	S.D	
At baseline	122.16	9.19	119.56	9.42	0.16
At 10 minutes	117.80	8.91	117.15	8.61	0.7
At 20 minutes	113.65	9.37	114.47	8.51	0.64
At 30 minutes	109.88	9.52	112.29	8.35	0.17
At 40 minutes	105.14	10.33	109.35	8.51	0.024*
At Intubation	96.00	10.11	99.35	9.06	0.08*
At 10 minutes post-intubation	111.88	7.99	108.04	9.07	0.023*
At 20 minutes post-intubation	110.78	7.71	108.44	9.14	0.16
At 30 minutes post-intubation	108.27	8.94	108.18	9.59	0.96
At 40 minutes post-intubation	105.92	9.79	108.73	11.45	0.18

In Table 4, the difference in mean DBP between IV and IN groups at different time intervals was shown. Of them, only at 40 minutes, at 10 minutes post-intubation, at 30 minutes post-intubation, and at 40 minutes post-intubation, the mean DBP difference between the groups was found to be significant.

**Table 4:** Difference in DBP between Intranasal and Intravenous DBP - diastolic blood pressure S.D - standard deviation

DBP	Intravenous		Intranasal		P value
	Mean	S.D	Mean	S.D	
At baseline	79.57	9.44	76.76	8.39	0.11
At 10 minutes	75.02	8.06	73.60	7.59	0.35
At 20 minutes	71.02	7.44	70.87	7.51	0.92
At 30 minutes	68.04	6.57	68.98	7.32	0.49
At 40 minutes	64.00	6.54	67.20	6.76	0.015*
At Intubation	59.06	4.77	60.55	5.09	0.13
At 10 minutes post-intubation	70.59	4.03	68.29	7.07	0.04*
At 20 minutes post-intubation	68.31	4.65	69.02	6.37	0.52
At 30 minutes post-intubation	64.98	5.62	68.44	6.74	0.005*
At 40 minutes post-intubation	62.82	5.51	68.22	7.61	0.0001*

**Figure 1:**
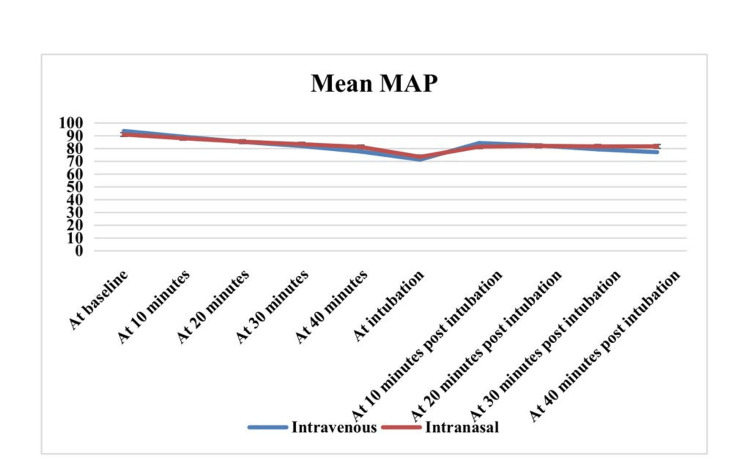
Difference in MAP between Intravenous and Intranasal groups MAP - mean arterial pressure

In Table 5 and Figure 1, the difference in mean MAP between IV and IN groups at different time intervals was shown. Of them, only at 40 minutes, at 10 minutes post-intubation, and at 40 minutes post-intubation, the mean MAP difference between the groups was found to be significant.

**Table 5:** Difference in MAP between Intranasal and Intravenous MAP - mean arterial pressure S.D - standard deviation

MAP	Intravenous		Intranasal		P value
	Mean	S.D	Mean	S.D	
At baseline	93.76	8.77	91.03	8.15	0.09
At 10 minutes	89.28	7.71	88.12	7.33	0.43
At 20 minutes	85.23	7.33	85.41	7.27	0.91
At 30 minutes	81.99	6.99	83.42	7.12	0.29
At 40 minutes	77.71	7.13	81.25	6.83	0.01*
At Intubation	71.37	6.01	73.48	5.93	0.07
At 10 minutes post-intubation	84.35	4.49	81.54	7.21	0.012*
At 20 minutes post-intubation	82.47	4.68	82.16	6.67	0.78
At 30 minutes post-intubation	79.411	5.82	81.68	7.39	0.083
At 40 minutes post-intubation	77.19	6.39	81.72	8.47	0.003*

## Discussion

For anesthesiologists, lowering the laryngoscopic stress reaction is a major concern. Dexmedetomidine used before surgery has been proven to decrease the laryngoscopic stress response. IN dexmedetomidine is becoming more used as a premedication nowadays in addition to IV, particularly among the pediatric population. We looked at how dexmedetomidine was administered intravenously and intranasally on fluctuations in hemodynamics during endotracheal intubation.

In terms of age and gender, both groups in our study were comparable. In group A IV dexmedetomidine compared to group B IN dexmedetomidine, the HR was substantially greater at baseline in group A. But in both groups, HR did not significantly differ post-intubation. The MAP has a similar pattern to the SBP and DBP, with the exception of baseline, 10 min after intubation, and 40 min after intubation.

Laryngeal intubation during GA causes noxious stimulation that significantly raises HR and MAP. This results from sympathetic activation and a rise in the amounts of catecholamines in the blood [8]. Changes in hemodynamics were studied for the first time by Raid and Brace during the laryngoscopy procedure. The reaction begins after five seconds of laryngoscopy, reaches its peak after one to two minutes, and then recovers to normal levels after 5 to 10 minutes [9].

An appropriate sympatholytic medication was needed to stop this sympathetic activity. No one class of pharmacological drugs, including opioids (fentanyl), adrenergic blockers (esmolol), vasodilators (sodium nitroprusside), and local anesthetics, can effectively reduce these hemodynamic changes. The unusual sedative, hypnotic, anxiolytic, sympatholytic, antisecretory, and analgesic characteristics of dexmedetomidine, a centrally acting alpha 2 agonist, make a popular choice in ICUs [10,11]. It does not produce respiratory depression, but it does have the unusual pharmacological effect of sedating the patient while they are awake. It is responsible for producing dose-dependent cooperative sedation, which enables early communication and early postoperative neurological assessment. Atipamizole, a medicine that dexmedetomidine has to counteract its sedative effects, works by raising noradrenaline turnover in the brain [12,13]. As a result of all of these unique qualities, dexmedetomidine has gained popularity as a preferred premedication agent. Presynaptic central alpha 2 receptor which is present in locus ceruleus, dexmedetomidine inhibits the release of noradrenaline and produces drowsiness and hypnosis [14,15]. The postsynaptic alpha 2 receptor, which inhibits tachycardia and hypertension, mediates the sympatholytic effect of dexmedetomidine. Because of the sympatholytic effect of dexmedetomidine, both IV and IN administrations of the drug were effective in lowering the laryngoscopic stress responses that were seen in this study.

According to a study by Niyogi et al. [7], it was discovered that preoperative IN dexmedetomidine (0.5g/kg) and preoperative IV dexmedetomidine infusion (1g/kg) had equal effects on the prevention of laryngeal intubation stress responses. The laryngoscopic stress reactions were successfully reduced by dexmedetomidine administered IV and IN, without noticeably raising BP and HR. For all of the hemodynamic measures before and during laryngeal intubation, the HR, SBP, DBP, and MAP of both groups were maintained within acceptable ranges (20% of baseline values).

Bon Sebastian et al. investigated the effectiveness of IV dexmedetomidine and NS in reducing the responsiveness of the patient's hemodynamics to the invasive procedures of laryngoscopy and endotracheal intubation. Dexmedetomidine significantly reduces HR and MAP compared to NS, according to the intergroup comparison [3].

This study's findings show that dexmedetomidine can be utilized as a premedication to decrease hemodynamic surges during endotracheal intubation with more or less the same efficacy via IN and IV routes. This result can be attributable to the fact that both IV and IN dexmedetomidine stop central catecholamine levels from rising.

## Conclusions

Preoperative dexmedetomidine has a proven track record of reducing laryngoscopy-induced stress responses. In addition to the IV, the availability of IN dexmedetomidine is gaining popularity as a premedication. In this study, it is concluded that both IV and IN dexmedetomidine when administered 40 minutes before induction are equally effective in attenuating hemodynamic surges during laryngoscopy and intubation. Hence, like IV dexmedetomidine even IN dexmedetomidine can be used as a safer alternative in attenuating hemodynamic responses.
